# Associations between exposure to digital food marketing and food consumption in adolescence: a cross-sectional study in an emerging country

**DOI:** 10.1186/s12889-025-24443-z

**Published:** 2025-09-30

**Authors:** Gastón Ares, Lucía Antúnez, Florencia Alcaire, Virginia Natero, Vanessa Gugliucci, Leandro Machín, Carolina de León, Tobias Otterbring

**Affiliations:** 1 Sensometrics & Consumer Science, Facultad de Química, Instituto Polo Tecnológico de Pando, Universidad de la República, By Pass de Rutas 8 y 101 s/n, CP 91000 Pando, Uruguay; 2 Centro de Investigación Básica en Psicología, Facultad de Psicología, Universidad de la República, Tristán Narvaja 1674, CP 11200 Montevideo, Uruguay; 3 Escuela de Nutrición, Universidad de la República, Montevideo, Uruguay; 4 División Salud, Intendencia de Montevideo, Montevideo, Uruguay; 5 School of Business and Law, Department of Management, University of Agder, Universitetsveien 17, 4630 Kristiansand, Norway

**Keywords:** Marketing, Digital marketing, Social media, Food environment, Adolescence

## Abstract

**Background:**

Evidence regarding the link between digital food marketing and eating habits is lacking in the majority world, i.e., the world regions where most people live. This study sought to investigate (i) self-reported exposure to digital food marketing, (ii) associations between such exposure and socio-demographic characteristics, and (iii) associations between exposure and food consumption frequency among adolescents in a Latin American country (Uruguay).

**Methods:**

A sample of adolescents aged between 11 and 19 years attending 29 public and 10 private high schools (*n* = 1542) was obtained through a cross-sectional survey using a stratified, two-stage cluster probability-based sampling approach. Participants filled out closed and open-ended questions about exposure to digital food marketing, food consumption frequency, social media usage, and socio-demographic characteristics. The data were analysed through descriptive statistics and ordinal logistic regressions.

**Results:**

Almost 90% of participants reported having seen a food or beverage advertisement on digital media in the week prior to the survey, with more than 70% of participants recalling advertisements of fast food, soft drinks, and savoury snacks. Age, socio-economic status, or total social media use did not predict exposure frequency of digital food marketing but females (vs. males) reported higher exposure. Exposure to advertisements of fast food or ultra-processed products on social media or websites and total social media use typically predicted higher reported consumption frequency of such categories. However, exposure to digital food marketing did not predict consumption frequency of fruits, vegetables, meats, or fish, although total social media use predicted lower consumption frequency of fruits and vegetables.

**Conclusions:**

Mere recall of exposure to digital food marketing and total social media use were associated with higher consumption frequency of ultra-processed products and fast food. These findings underscore the need to introduce comprehensive mandatory policies to reduce adolescent exposure to digital food marketing featuring unhealthy foods.

**Supplementary Information:**

The online version contains supplementary material available at 10.1186/s12889-025-24443-z.

## Background

Food marketing of unhealthy foods is one of the commercial determinants of health [1, 2]. Through a hierarchy of sequential effects, exposure to food marketing influences food-related attitudes, increases purchases and consumption, and, in the long term, impacts health-related outcomes [3]. With the advent of the internet, digital marketing has become one of the most prevalent components of the marketing mix of food companies [4–6]. It can be defined as any “*promotional activity*,* delivered through a digital medium*,* that seeks to maximize impact through creative and/or analytical methods*” [7].

Digital marketing has a series of characteristics that make it potentially more persuasive than other forms of off-line marketing. Firstly, digital media are ubiquitous and not confined to specific settings, providing opportunities to reach audiences anytime and anywhere [8]. It can be tailored to the specific characteristics of users through audience segmentation strategies based on data analytics [9]. Digital media also enables interactions with users, endorsement of brand messages, as well as embedding advertisements within entertainment content, which increases persuasiveness [8, 10–12].

Exposure to digital food marketing is highly relevant for adolescents as they are avid users of digital media [13, 14]. Susceptibility to the persuasive effects of unhealthy food marketing is particularly high in adolescence due to neurodevelopmental mechanisms that heighten sensitivity to rewards, reduce inhibitory control, and encourage risky behaviours [15, 16]. In addition, adolescents are in a key stage of their identity development and are highly susceptible to social pressure, wanting to conform to social norms around food consumption [17, 18]. Increased autonomy and agency and reduced parental control and guidance during this life period may also increase vulnerability to the persuasive effects of marketing [19].

Adolescents across the globe have been reported to be frequently exposed to digital marketing of unhealthy foods, especially fast food and ultra-processed products such as sweetened beverages, chocolates and confectionary, savoury snacks and breakfast cereals [20–28]. However, knowledge on the effect of exposure to digital food marketing on the eating habits of adolescents is still limited [29, 30]. A handful of observational studies conducted in Australia, Belgium, and the UK have associated self-reported exposure to digital marketing, both in general and on specific platforms, with increased consumption of unhealthy foods and drinks [22, 23, 25, 31, 32]. In addition, a recent experimental study conducted in a Latin American country has shown that exposure to digital marketing of unhealthy foods leads to increased advertisement and brand recall [33].

Additional information on the nature, extent, and impact of digital food marketing is needed to strengthen public policies to protect adolescents [9]. In particular, evidence on the associations between exposure to digital food marketing and eating habits is still lacking in the majority world, i.e., the regions of the world where most of the population live [34]. Therefore, this study aimed at expanding current knowledge by examining exposure to digital marketing of ultra-processed products and fast food among adolescents in Uruguay, a high-income Latin American country.

The present cross-sectional observational study was part of a larger project on Uruguayan adolescents’ exposure to digital food marketing. Uruguay has one of the highest prevalence of overweight and obesity among adolescents in the region: 33.6% among adolescents aged 13–17 years [35]. Unhealthy diets, and particularly consumption of ultra-processed products, has been identified as a relevant behavioural risk factor contributing to adolescent overweight and obesity [35–38]. Internet access is widespread among Uruguayan adolescents. According to a nationally representative survey conducted in 2019, 94% of the adolescents between 14 and 19 years had daily access to the internet, and they all had at least one social media account [39]. At the time of the study, the country did not have any regulations or guidelines on food marketing on mass media, including digital food marketing.

The objectives of the study were to: (i) estimate self-reported exposure to digital food marketing among Uruguayan adolescents, (ii) analyse associations between such exposure and socio-demographic characteristics, and (iii) analyse associations between said exposure and food consumption frequency.

## Methods

### Participants

A stratified, two-stage cluster probability-based sampling approach was used to obtain a sample of adolescents in grades 1–6 (typically aged 12 and 18 years) attending public and private high schools in Montevideo, the capital city of Uruguay. The sampling frame included all the public (*n* = 77) and private (*n* = 94) high schools in Montevideo, registered at the National Administration of Public Education.

The first stage consisted of selecting high schools as primary sampling units using probability proportional to size. High schools were stratified by type (public vs. private) to ensure the sample reflected the local distribution of students. The probability of selection of each public/private high school was proportional to the proportion of students in the corresponding type of high school. A sample of 39 high schools was obtained, composed of 29 public and 10 private schools. The research team contacted each school to obtain authorization from the principal and to request information about the number of classes in each grade and the corresponding number of students. All chosen schools agreed to participate.

The second sampling stage consisted of selecting classes within each school. One class within each grade was randomly selected. All students attending school the day the questionnaires were distributed were eligible for participation. A total of 3974 students were initially selected to participate in the survey.

### Questionnaire

A questionnaire was developed by the research team based on a previous qualitative study on adolescents’ experiences with digital food marketing [27], and questionnaires from published studies [22, 25, 31]. It included four main sections: (i) exposure to digital food marketing, (ii) food consumption frequency, (iii) social media usage, and (iv) socio-demographic characteristics.

Frequency of exposure to digital food marketing was assessed using the question: *Have you seen advertisements of foods and beverages on social media or websites in the last week?* The following response options were available: ‘Yes, more than once a day’, ‘Yes, once a day’, ‘Yes, several times a week’, ‘Yes, once in the week’ and ‘No, I haven’t seen any advertisement’. Frequency of exposure was then recoded into the following three categories: ‘No exposure’, ‘Less than once a day’, ‘Once a day or more’. Then, participants indicated their spontaneous recall of food and beverage advertisements on social media by answering the following open-ended question: *What advertisements of food and beverages do you recall seeing on social media*? (data not analysed in the present work). Exposure to advertisements on specific social media was assessed through the following question: *In which of the following media do you remember seeing any food or beverage advertisements in the last week*? For each of the following social media, participants had to indicate Yes or No: Instagram, TikTok, Facebook, YouTube, Snapchat, Twitter, Twitch, Website browsing. The final question of the section explored prompted recall of advertisements of specific foods: *Do you remember seeing any advertisement of the following food or beverages on social media or websites in the last week*? Participants had to answer ‘Yes’ or ‘No’ for each of the following products: (i) soft drinks, (ii) energy drinks, (iii) flavoured water, (iv) bottled juices or powdered drinks, (v) cookies or crackers, (vi) *alfajores* [traditional product composed of two soft biscuits, joined by a sweet filling, usually covered in chocolate], (vii) flavoured milk, yogurt or milk desserts, (viii) chocolates or confectionary, (ix) ice-cream, (x) cold cuts or sausages (e.g., ham, salami, sausages, hot dogs), xi) breakfast cereals or cereal bars, xii) bakery products (e.g., croissants, donuts, pastries), xiii) savoury snacks (e.g., potato chips, Doritos, Cheetos), xiv) Condiments (e.g., ketchup, mustard, or mayonnaise), xv) soups or broths, xvi) marmalades or *dulce de leche* [local type of sweetened condensed milk], xvii) food eaten in fast food restaurants (e.g., burgers, pizza, French fries).

In the second section, participants were asked to indicate how often they ate a variety of foods the week prior to the survey (‘*How many days last week did you eat* (name of the food item)?): (i) fruits, (ii) vegetables, (iii) beef, chicken or pork, (iv) fish, (v) milk or cheese, (vi) soft drinks, (vii) energy drinks, (viii) flavoured water, iv) bottled juices or powdered drinks, x) cookies or crackers, xi) alfajores, xii) flavoured milk, yogurt or milk desserts, xiii) chocolates or candies, xiv) ice-cream, xv) cold cuts or sausages (e.g., ham, salami, sausages, hot dogs), xvi) breakfast cereals or cereal bars, xvii) bakery products (e.g., croissants, donuts, pastries), xviii) savoury snacks (e.g., potato chips, Doritos, Cheetos), xix) ketchup, mustard, or mayonnaise, xx) at a fast food restaurant (e.g., burgers, pizza, French fries). To capture take-away consumption, the question on fast food consumption was phrased as ‘How many days last week did you eat at or order from a fast-food restaurant?’. The response options for all items were: ‘0 day’, ‘1 day’, ‘2–3 times’, ‘4–6 times’, ‘7 days’.

Self-reported daily social media time was measured using the question: *On a normal weekday*,* how much time do you use* (name of the social media)? Participants were asked to answer this question for eight media: Instagram, TikTok, Facebook, Twitter, YouTube, Snapchat, Twitch, Website browsing (e.g., Google). Participants were also asked about their time spent watching TV: *On a normal weekday*,* how much time do you watch TV (including cable TV*,* Netflix*,* Disney + or other platforms*)? For both questions the response options were: ‘I don’t use’, ‘Less than 15 minutes’, ‘15 to 30 minutes’, ‘30 min to 1 hour’, ‘1 to 2 hours’, ‘2 to 3 hours’, ‘3 to 4 hours’, ‘More than 4 hours’. Responses were recoded considering the middle point of the range (e.g., 15 to 30 min was recoded to 22.5 min). The response option ‘More than 4 hours’ was recoded as 240 min (4 h). Participants’ total exposure to digital media combined (in hours) was calculated by summing up the time spent on the seven digital media (Instagram, TikTok, Facebook, Twitter, YouTube, Snapchat, Twitch, Website browsing).

The last section focused on socio-demographic information. Participants were asked to indicate their gender (male, female, other), age, and neighbourhood of residence. Socio-economic status was estimated using the score of the neighbourhood of residence in the national socio-economic status index, which ranges from 0 to 14 (Centro de Investigaciones Económicas, 2023) [40]. The scores associated to each socio-economic status were: <4 low socio-economic status, *≥* 4 and < 11 medium socio-economic status, ≥ 11 high socio-economic status.

The English version of the questionnaire is shown in Supplementary Material Fig. 1. The questionnaire was pilot tested with a convenience sample of 17 adolescents of different socio-economic status. After they completed the questionnaire, they were asked if they had faced any challenge or difficulty. As they all answered negatively, no changes were introduced before data collection.

### Data collection

Paper-based questionnaires were delivered to the high schools by members of the research team. The staff of each high school oversaw distributing the questionnaires to all the students in the selected classes. Participants not attending classes the day the questionnaires were distributed did not participate in the study.

The questionnaires were self-administered and completed at participants’ homes. They included an introductory letter addressed to parents, explaining about the purpose of the survey and providing contact information if they had any questions. Informed written consent from one of the parents (or an adult in charge of the adolescent) and written assent from the adolescent were required. For adolescents aged 18 years or older, parental informed consent was not required. Adolescents were instructed to give the questionnaires to their parents first. The introductory letter emphasized that parents should first sign the informed consent form before passing the questionnaire to their child to provide assent and complete it. Both parents and adolescents were informed that the study was related to social media, but no specific mention to digital marketing or food was included in the information sheet to minimize potential response biases. No compensation for participation was provided.

After the questionnaires had been collected from participants, school staff contacted the research team to pick them up. Data collection was performed between April and July 2024. After data analysis was finalized, high school principals, participants, and their parents were sent information sheets summarizing the key results of the study.

### Data analyses

All data analyses were performed in R software [41]. Descriptive statistics were used to summarize results from multiple choice questions. Average, median and standard deviation were calculated for total exposure to digital media. Participants with missing data (*n* = 24) or whose self-reported gender identities (*n* = 9) differed from males or females were not considered in the analyses given the small sample size and, hence, insufficient statistical power to detect ‘typical’ effect sizes in the published literature.

An ordinal logistic regression model was used to analyse associations between self-reported exposure to digital food marketing and socio-demographic variables. The model was run considering self-reported frequency of exposure in the week prior to the survey (‘None’, ‘Less than once a week’, ‘Once a week or more’) as the dependent variable, whereas the following individual characteristics were considered as independent variables: gender (male vs. female), age (range 11–14 years old vs. 15–19 years old), socio-economic status (low vs. high and low vs. medium), total social media use (in hours), and TV watching time (in hours).

The association between consumption frequency of ultra-processed products and fast food and self-reported exposure to digital marketing was assessed using an ordinal logistic regression model. For each food category included in the questionnaire, a separate ordinal logistic regression model was run considering consumption frequency (‘0 days’, ‘1 day’ and ‘2–3 days’, ‘4–6 days’ and ‘7 days’) as the dependent variable and the following independent variables: self-reported exposure to an advertisement of the category on social media (Yes vs. No) and total social media use (in hours). The models controlled for gender, age range, socio-economic status, and TV watching time (in hours). Similar models were run to analyse the relationship between self-reported exposure to digital food marketing (Yes vs. No) on consumption frequency of natural foods (fruits, vegetables, meat, and fish).

The proportional odds assumption of the ordinal logistic regression models was assessed using Lipsitz goodness of fit test and Pulkstenis-Robinson chi-squared test [42]. These tests were conducted using the package generalhoslem in R [43].

## Results

A total of 1542 adolescents, aged between 11 and 19 years (M = 14.3, SD = 1.8), completed the questionnaire. The number of completed questionnaires corresponded to 38.8% of the total number of students in the selected classes. However, the actual response rate was most likely somewhat higher as high school staff did not report the number of students who were sick or absent for other reasons. As such, our effective response rate is likely an underestimation of the actual response rate.

The sample comprised 56.2% of females, 42.5% of males, and 0.6% WHO identified with other gender identities. Approximately 6 out of 10 participants were 11–14 years old and lived in neighbourhoods of medium socio-economic status (Table 1). The great majority of participants (99.5%) reported using at least one digital media. Website browsing, YouTube, Instagram, and TikTok were the most popular media, being used by more than 80% of the participants. Although the minimum age for signing up to Instagram and TikTok is 13 years [44, 45], 72.9% and 65.6% of the participants younger than this age reported using these platforms, respectively. Total daily use of digital media accounted for an average of 5.5 h and a median of 5.0 h (SD = 3.2).

**Table 1:** Characteristics of the participants (*n* = 1542)

Characteristic	Number of participants	Percentage of participants (%)
Gender		
Female	866	56.2
Male	655	42.5
Other	9	0.6
No response	12	-
Age		
11-14	909	58.9
15-19	619	40.1
No response	14	-
Socio-economic status		
Low	214	13.9
Medium	938	60.8
High	364	23.6
No response	26	-
Digital media use (*)		
Any digital media	1535	99.5
Website browsing	1438	99.3
YouTube	1335	87.3
Instagram	1273	84.0
TikTok	1226	80.1
Twitter	434	28.3
Twitch	322	21.0
Facebook	148	9.7
Snapchat	113	7.4

Participants with missing data were not considered in the calculation of percentages. Socio-economic status was estimated based on the score of the neighbourhood of residence in the national socio-economic status index [40]

### Self-reported exposure to digital food marketing

The percentage of participants WHO reported seeing a food or beverage advertisement on digital media in the week prior to the survey corresponded to 87.6%, with 39.1% reporting daily exposure (Table 2). Instagram, YouTube, and TikTok were the platforms where participants reported being most frequently exposed to food and beverage advertisements. As shown in Table 2, the percentage of participants WHO reported seeing an advertisement on these platforms ranged from 63.5 to 72.6%. Less than 10% of the participants recalled seeing food and beverage advertisements on Twitch, Facebook, or Snapchat.

**Table 2:** Exposure to food and beverage advertisements in digital media, presented as number and percentage of participants reporting having seen food and beverage advertisements the week prior to the survey (n=1509).

Type of exposure to food and beverage advertisements in digital media	Number of participants	Percentage of participants (%)
Frequency of exposure to advertisements of food and beverages on social media or websites in the week prior to the survey		
No exposure	187	12.4
At least once a week	590	48.5
At least once per day	732	39.1
Exposure to advertisements of food and beverages in the previous week on specific digital media in the week prior to the survey		
Instagram	1095	72.6
YouTube	1011	67.0
TikTok	958	63.5
Website browsing	456	30.2
Twitter	196	13.0
Twitch	136	9.0
Facebook	74	4.9
Snapchat	10	0.7
Exposure to advertisements of spemcific products on social media or websites in the week prior to the survey		
Fast food	1218	80.7
Soft drinks	1087	72.0
Savoury snacks	1063	70.4
Alfajores	703	46.6
Ice-cream	636	42.1
Energy drinks	635	42.1
Flavoured water	565	37.4
Chocolates and confectionary	559	37.0
Bakery products	494	32.7
Condiments	480	31.8
Cold cuts or sausages	449	29.8
Cookies or crackers	408	27.0
Flavoured milk, yogurt or dairy desserts	364	24.1
Bottled juices or powdered drinks	324	21.5
Breakfast cereals or cereal bars	263	17.4
Marmalades or dulce de leche	222	14.7
Soups or broths	184	12.2

Participants with missing data (*n* = 24) or whose self-reported gender identities (*n *= 9) differed from males or females were not included

When participants were prompted to recall advertisements of specific product categories on digital media, more than 7 of 10 participants reported seeing advertisements of fast food, soft drinks, and savoury snacks (Table 2). On the contrary, less than 2 of 10 recalled seeing advertisements of breakfast cereals and cereal bars, marmalade or dulce de leche, and soups or broths.

### Associations of self-reported frequency of exposure to digital food marketing with socio-demographic characteristics

As shown in Table 3, self-reported frequency of exposure to digital food marketing was not significantly associated with age, socio-economic status, total social media use (in hours) or TV watching time (hours). The only variable with a significant association was gender. The odds of reporting a higher frequency of exposure to digital food marketing was 21% higher for females than for males, holding constant all other variables.

**Table 3:** Results of the ordinal logistic regression analysing the association between socio-demographic characteristics and self-reported frequency of exposure to digital food marketing, expressed as odd-ratios with 95% confidence intervals and *p*-values

Characteristic	Odd-ratio	*p*-value
Gender		
Female	1	-
Male	**0.79 (0.64–0.95)**	**0.013**
Age range		
11–14	1	-
15–19	0.85 (0.70–1.04)	0.125
Socio-economic status		
Low	0.94 (0.60–1.17)	0.297
Medium	1.00 (0.79–1.27)	0.997
High	1	-
Total social media use (hours)	0.98 (0.95–1.01)	0.146
TV watching time (hours)	0.93 (0.85–1.01)	0.079

Participants with missing data (*n* = 24) or whose self-reported gender identities (*n* = 9) differed from males or females were not included in the model. The reference in the model was ‘No exposure to digital food marketing’ compared to ‘Less than once a week’ and ‘At least once a week’. Odd-ratios highlighted in bold were significant at 0.05. The proportional odds assumption was accepted according to Lipsitz goodness of fit test (0. 271) and Pulkstenis-Robinson chi-squared test (*p*-value = 0.537)

### Associations between exposure to digital food marketing and food consumption frequency

Table 4 shows results of the ordinal logistic regression models analysing the associations between recall of exposure to advertisements of specific product categories and consumption frequency. Self-reported exposure to an advertisement of ultra-processed products or fast food on social media or websites was significantly associated with increased likelihood of reporting higher consumption frequency of the category, while controlling for all other variables. The only exception was the category cookies and crackers, whose consumption frequency was not significantly associated with exposure to advertisements on social media or websites in the week prior to the survey (*p* = 0.988). For the rest of the food categories, the increase in the odds of reporting a higher consumption frequency ranged from 31% for fast food to 114% for energy drinks (Table 4).

**Table 4:** Results of the ordinal logistic regressions analysing the association between consumption frequency of specific ultra-processed product categories and self-reported exposure to an advertisement of the food category on digital media in the week prior to the survey and total social media use (hours), expressed as odd-ratios with 95% confidence intervals

Product category	Exposure to an advertisement in the week prior to survey (Yes vs. No)	Total social media use (hours)
Soft drinks	**1.53 (1.24–1.88)**	**1.07 (1.05–1.11)**
Energy drinks	**2.14 (1.67–2.76)**	**1.09 (1.05–1.13)**
Flavoured water	**1.81 (1.46–2.24)**	1.02 (0.99–1.05)
Bottled juices or powdered drinks	**1.32 (1.05–1.67)**	**1.05 (1.02–1.08)**
Savoury snacks	**1.82 (1-48-2.26)**	**1.07 (1.04–1.10)**
Bakery products	**1.41 (1.16–1.72)**	**1.05 (1.02–1.08)**
Cookies or crackers	1.18 (0.95–1.45)	1.02 (0.99–1.05)
Alfajores	**1.64 (1.35–1.98)**	**1.07 (1.04–1.10)**
Breakfast cereals or cereal bars	**1.71 (1.30–2.23)**	1.01 (0.98–1.05)
Flavoured milk, yogurt or dairy deserts	**1.90 (1.53–2.36)**	**1.05 (1.02–1.08)**
Ice-cream	**1.35 (1.09–1.68)**	1.00 (0.91–1.26)
Chocolates or confectionary	**1.86 (1.53–2.27)**	**1.05 (1.00-1.08)**
Cold cuts and sausages	**1.37 (1.11–1.69)**	**1.03 (1.00-1.06)**
Ketchup or mayonnaise	**1.49 (1.22–1.81)**	**1.05 (1.02–1.09)**
Fast food	**1.31 (1.00-1.73)**	**1.07 (1.03–1.10)**

Participants with missing data (*n* = 24) or whose self-reported gender identities (*n* = 9) differed from males or females were not included in the model. The reference in the model for consumption frequency was ‘0 days’ compared to ‘1 day’, and ‘2–3 days’, ‘4–6 days’ and ‘7 days’. Odd-ratios highlighted in bold were significant at 0.05. The models controlled for gender, age range, socio-economic status and TV watching time. The *p*-values of the full models and results of the Lipsitz goodness of fit and Pulkstenis-Robinson chi-squared tests are shown in the Supplementary Material Table 1

For 12 of the 16 categories, total social media use was significantly associated with an increased likelihood of reporting higher consumption frequency. For each additional hour of social media use, the increase in the odds of reporting a higher consumption frequency of ultra-processed products or fast food ranged between 3% and 9%, after controlling for all other variables (Table 4). The four categories that did not follow this trend were flavoured water (*p* = 0.241), cookies or crackers (*p* = 0.150), breakfast cereals and cereal bars (*p* = 0.487), and ice-cream (*p* = 0.098).

As shown in Table 5, self-reported exposure to digital food marketing in the week prior to the survey was not significantly associated with consumption frequency of fruits, vegetables, meats, or fish. However, total social media use was significantly associated with a reduced likelihood of reporting higher consumption frequency of fruits and vegetables. For every hour increase in social media use, the odds of being more likely to report high consumption frequency of fruits decreased by 6%, whereas the likelihood of reporting high consumption frequency of vegetables decreased by 4%. For meats and fish, consumption frequency was not significantly associated with total social media use (Table 5).

**Table 5:** Results of the ordinal logistic regressions analysing the association between consumption frequency of specific food groups and self-reported exposure to digital food marketing in the week prior to the survey and total social media use (hours), expressed as odd-ratios with 95% confidence intervals.

Product category	Exposure to digital food marketing in the week prior to the survey (Yes vs. No)	Total social media use (hours)
Fruits	1.27 (0.80-2.00)	**0.94 (0.91–0.96)**
Vegetables	1.27 (0.81–2.01)	**0.96 (0.93–0.99)**
Meats	1.11 (0.70–1.76)	1.02 (0.99–1.05)
Fish	1.02 (0.63–1.70)	0.98 (0.95–1.01)

Participants with missing data (*n* = 24) or whose self-reported gender identities (*n* = 9) differed from males or females were not included in the model. The reference in the model for consumption frequency was ‘0 days’ compared to ‘1 day’, and ‘2–3 days’, ‘4–6 days’ and ‘7 days’. Odd-ratios highlighted in bold were significant at 0.05. The models controlled for gender, age range, socio-economic status and TV watching time. The *p*-values of the full models and results of the Lipsitz goodness of fit and Pulkstenis-Robinson chi-squared tests are shown in the Supplementary Material Table 2

## Discussion

The present study explored the role of exposure to digital food marketing among adolescents in Uruguay, a high-income Latin American country. Results showed frequent exposure to digital food marketing: almost 9 of 10 participants reported having seen a food and beverage advertisement on digital media in the week prior to the survey. This percentage is in the upper range of self-reported exposure reported in previous studies [22, 28].

Instagram, YouTube, and TikTok emerged as the media where participants most frequently reported seeing food and beverage advertisements. An in-depth analysis of marketing activities in these platforms can inform the development of regulations to reduce the persuasiveness of digital food marketing [9, 46]. In this sense, previous studies have reported that Instagram posts include a wide range of cues that trigger systematic and heuristic processing in an attempt to maximize persuasiveness, including interactions with users, videos and music, contests and raffles, price promotions, memes or pranks as well as references to specific characteristics of the products [47–52]. Recent studies have also explored how influencers promote unhealthy foods and beverages on YouTube and TikTok [53, 54]. However, an in-depth characterization of the prevalence and power of food and beverage advertisements on these platforms is lacking.

Advertisements of fast food, soft drinks, and savoury snacks were the most frequently recalled by participants, consistent with results from a previous qualitative study with Uruguayan adolescents [27]. Several studies conducted in different countries around the globe have also identified these categories as the most frequently advertised across different media, including social media platforms [21, 24, 26, 28, 55–57] and TV [58]. Further, these categories have been found to be frequently marketed to adolescents [59], and to contribute to the largest proportion of the expenditure on food marketing targeted at children and adolescents [60].

Self-reported frequency of exposure to digital food marketing was not significantly associated with participants’ age and socio-economic status. The lack of age differences is particularly concerning given that several social media platforms have an age requirement of 13 years [61]. Results from the present study indicate that many platforms lack adequate protection for children and adolescents, as participants below the minimum age requirement frequently reported using social media and seeing unhealthy food advertisements.

Total social media use was not significantly associated with self-reported frequency of exposure to digital food marketing. Although this result may seem counterintuitive, it can be explained by differences in the type and recognizability of marketing across various social media platforms. Some advertisements, such as those embedded in influencer content or native advertising, may be less easily identified as marketing, leading to underreporting. In addition, the large number of food and beverage advertisements typically found in digital media may dilute the overall association between total social media use and self-reported marketing exposure. Studies have shown that users are exposed to several advertisements of unhealthy foods and beverages per hour across social media platforms and websites [20, 26, 57].

Gender was the only socio-demographic variable with a significant association with self-reported frequency of exposure to digital food marketing. Females had greater odds of being frequently exposed to digital food marketing compared to males. This result differs from a previous study reporting similar exposure to digital food marketing for male and female adolescents in Canada [24]. Considering that gender has been reported to mediate the effect of marketing on food preferences [62], further research is needed to investigate gender differences in adolescents’ experiences with digital food marketing across countries and cultures.

Results from the present work contribute to the growing body of literature reporting associations between exposure to digital food marketing and eating habits [22, 23, 25, 31, 32]. Self-reported exposure to advertisements of fast food and ultra-processed products was associated with increased likelihood of reporting high consumption frequency. As far as can be ascertained, this is the first study to report associations between exposure to advertisements and consumption frequency of specific product categories. Previous studies have found general associations between exposure to digital food marketing and consumption of unhealthy foods and beverages [22, 23, 25, 31, 32]. In the present work, mere self-reported exposure to digital food marketing was associated with higher consumption frequency of ultra-processed products and fast food, as postulated by the Hierarchy of Unhealthy Food Promotion Effects [3, 63]. This extends results from the study conducted by Baldwin et al., who reported that intake of unhealthy foods was only associated with interaction with advertisements and not with passive exposure [32].

The present study cannot establish causality between exposure to digital food marketing and eating habits. Higher consumption frequency of a specific product category can lead to attentional bias, making adolescents more likely to attend to and recall advertisements of products they like and frequently consume [64]. In addition, targeting algorithms in social media can trigger more frequent exposure to advertisements of specific products because of higher user interest [9]. Reverse causality (i.e., higher consumption frequency triggering higher exposure to advertisements) is also worrying from a public health perspective, as it can contribute to reinforce unhealthy food preferences and social norms around consumption of fast food [17].

The present work shows an association between total social media use and consumption frequency of fast food and several categories of ultra-processed products, while controlling for self-reported exposure to advertisements of the specific category. This result may be explained by exposure to food advertisements without conscious awareness, as suggested by the Reactivity to Embedded Food Cues in Advertising Model [65]. Adolescents may process advertisements implicitly, creating positive emotional and symbolic associations with products [46]. In this sense, exposure to digital food marketing and user-generated content of ultra-processed products and fast food may contribute to create social norms around food, reinforcing adolescents’ unhealthy dietary habits [25]. Results from the current research also show that total social media use is associated with reduced consumption frequency of fruits and vegetables. Further research should delve into how social norms around food are created in adolescence and whether social media use may make adolescents prioritize heavily advertised unhealthy food options characterized by a high reward value (e.g. ultra-processed products), while downplaying the importance of healthier alternatives.

Together, the findings from this study highlight the need to reduce adolescent exposure to digital food marketing featuring unhealthy foods. Considering that social media platforms have not adopted restrictions on unhealthy food marketing [66], mandatory policies are needed. This regulatory approach is recommended by the World Health Organization [9, 67], and supported by a systematic review suggesting that marketing regulation has the potential to reduce purchases of foods high in sugars, saturated fat, and sodium [68]. Given that adolescents are influenced by digital food marketing [69], even when it is not specifically targeted at them, the focus should arguably be placed on the general population instead of a sole focus on vulnerable consumer segments. A total ban of digital food marketing featuring unhealthy food seems warranted [70]. Empowering adolescents on the topic can also contribute to improve their ability to resist the persuasiveness of digital food marketing [71], and may increase political will for action [72, 73]. An interesting avenue for further research, therefore, is the use of co-creation for developing strategies to achieve this objective [74].

### Strengths and limitations

The main strength of this study is its novelty, as it addressed an emerging topic on an underrepresented population in the food marketing literature [29, 30]. From a methodological point of view, the study has several strengths related to the large sample size, the recruitment method, and the specificity of the questions on digital food marketing recall. Another strength was the school response rate, with all selected high schools agreeing to participate in the study.

Several limitations should also be noted. First, the study was based on self-reported data of exposure to food and beverage advertisements. Thus, the study only captured the advertisements adolescents attended to and recalled. Actual exposure to advertisements may be underreported considering that advertisements may be embedded in entertainment or media content generated by celebrities or influencers, which can be difficult to distinguish for adolescents [75]. Additionally, social media use was assessed only for a normal weekday, and not for normal weekend days, when adolescents generally have more free time. This methodological choice may have led to an underestimation of total media use. Adolescents also self-reported their consumption frequency, which could be influenced by social desirability bias. Indeed, a relevant share of adolescents tends to underreport their energy intake [76, 77]. The order of the survey questions may have introduced response bias, as participants were asked about their food consumption after responding to questions about their marketing exposure. Finally, the study did not control for exposure to other types of marketing, such as TV or out-of-home advertising.

## Conclusions

The present work sheds light on the extent to which adolescents in an emerging Latin American country report being exposed to digital food marketing featuring unhealthy foods and beverages. It makes a significant contribution to the literature by reporting associations between self-reported exposure to advertisements on social media and websites, social media use, and consumption frequency of fast food and ultra-processed products. The study is the first to report associations between exposure to advertisements and consumption of specific product categories. Although causality cannot be established, results are consistent with the Hierarchy of Unhealthy Food Promotions Effects and Reactivity to Embedded Food Cues in Advertising Models. The findings stress the need to implement regulatory approaches to reduce exposure to digital marketing of unhealthy foods and beverages and enable adolescents to adopt and maintain healthy eating habits.

## Supplementary Information

Supplementary Material 1

## Data Availability

The data that support the findings of this study are available from the authors upon reasonable request.

## References

[1] Mialon M. An overview of the commercial determinants of health. Global Health. 2020;16:1–7. 32807183 10.1186/s12992-020-00607-x PMC7433173

[2] Kickbusch I, Allen L, Franz C. The commercial determinants of health. Lancet Glob Health. 2016;4:e895–6. 27855860 10.1016/S2214-109X(16)30217-0

[3] Kelly B, King L, Chapman K, Boyland E, Bauman AE, Baur LA. A hierarchy of unhealthy food promotion effects: identifying methodological approaches and knowledge gaps. Am J Public Health. 2015;105:e86–95. 25713968 10.2105/AJPH.2014.302476 PMC4358159

[4] Felix R, Rauschnabel PA, Hinsch C. Elements of strategic social media marketing: A holistic framework. J Bus Res. 2017;70:118–26.

[5] Nielsen. Global annual marketing report 2022. New York; 2022.

[6] Vollero A, Valentini C. Social media and consumer power. Opportunities and challenges for digital marketing activities. In: Niininen O, editor. Contemporary issues in digital marketing. New York, NY: Routledge; 2022. pp. 105–16.

[7] World Cancer Research Fund International. Building momentum: lessons on implementing robust restrictions of food and non-alcoholic beverage marketing to children. World Cancer Research Fund International; 2020.

[8] Appel G, Grewal L, Hadi R, Stephen AT. The future of social media in marketing. J Acad Mark Sci. 2020;48:79–95. 32431463 10.1007/s11747-019-00695-1 PMC7222052

[9] World Health Organization. Tackling food marketing to children in a digital world: trans-disciplinary perspectives Children’s rights, evidence of impact, methodological challenges, regulatory options and policy implications for the WHO European Region. 2016.

[10] Kannan PK, Li H. Digital marketing: A framework, review and research agenda. Int J Res Mark. 2017;34:22–45.

[11] Buchanan L, Kelly B, Yeatman H, Kariippanon K. The effects of digital marketing of unhealthy commodities on young people: A systematic review. Nutrients. 2018;10:1–19. 10.3390/nu10020148 PMC5852724 29382140

[12] Montgomery K, Chester J, Nixon L, Levy L, Dorfman L. Big data and the transformation of food and beverage marketing: undermining efforts to reduce obesity? Crit Public Health. 2019;29:110–7.

[13] Valkenburg PM, Piotrowski JT. Plugged in: how media attract and affect youth. New Haven, CT: Yale University Press; 2017.

[14] Shankleman M, Hammond L, Jones FW. Adolescent social media use and Well-Being: A systematic review and thematic Meta-synthesis. Adolesc Res Rev. 2021;6:471–92.

[15] Lowe CJ, Morton JB, Reichelt AC. Adolescent obesity and dietary decision making—a brain-health perspective. Lancet Child Adolesc Health. 2020;4:388–96. 32164832 10.1016/S2352-4642(19)30404-3

[16] Van Leijenhorst L, Zanolie K, Van Meel CS, Westenberg PM, Rombouts SARB, Crone EA. What motivates the adolescent?? Brain regions mediating reward sensitivity across adolescent?ce. Cereb Cortex. 2010;20:61–9. 19406906 10.1093/cercor/bhp078

[17] Buijzen M, Van Reijmersdal EA, Owen LH. Introducing the PCMC model: an investigative framework for young people’s processing of commercialized media content. Communication Theory. 2010;20:427–50.

[18] Pechmann C, Levine L, Loughlin S, Leslie F. Impulsive and Self-Conscious: adolescents’ vulnerability to advertising and promotion. J Public Policy Mark. 2005;24:202–21.

[19] Neufeld LM, Andrade EB, Ballonoff Suleiman A, Barker M, Beal T, Blum LS, et al. Food choice in transition: adolescent autonomy, agency, and the food environment. Lancet. 2022;399:185–97. 34856191 10.1016/S0140-6736(21)01687-1

[20] van der Bend DLM, Jakstas T, van Kleef E, Shrewsbury VA, Bucher T. Adolescents’ exposure to and evaluation of food promotions on social media: a multi-method approach. Int J Behav Nutr Phys Activity. 2022;19:74. 10.1186/s12966-022-01310-3 PMC9235222 35761362

[21] Elliott C, Truman E, Black JE. Tracking teen food marketing: participatory research to examine persuasive power and platforms of exposure. Appetite. 2023;186:106550. 37019155 10.1016/j.appet.2023.106550

[22] Gascoyne C, Scully M, Wakefield M, Morley B. Food and drink marketing on social media and dietary intake in Australian adolescents: findings from a cross-sectional survey. Appetite. 2021;166:105431. 34062174 10.1016/j.appet.2021.105431

[23] Evans R, Christiansen P, Masterson T, Pollack C, Albadri S, Boyland E. Recall of food marketing on videogame livestreaming platforms: associations with adolescent diet-related behaviours and health. Appetite. 2023;186:106584. 37127245 10.1016/j.appet.2023.106584

[24] Amson A, Pauzé E, Remedios L, Pritchard M, Potvin Kent M. Adolescent exposure to food and beverage marketing on social media by gender: a pilot study. Public Health Nutr. 2023;26:33–45. 36321517 10.1017/S1368980022002312 PMC11077454

[25] Qutteina Y, Hallez L, Raedschelders M, De Backer C, Smits T. Food for teens: how social media is associated with adolescent eating outcomes. Public Health Nutr. 2022;25:290–302. 10.1017/S1368980021003116 PMC8883778 34325764

[26] Potvin Kent M, Bagnato M, Remedios L, Soares Guimarães J, Gillis G, Soto C, et al. Child and adolescent exposure to unhealthy food marketing across digital platforms in Canada. BMC Public Health. 2024;24:1740. 38951838 10.1186/s12889-024-19094-5 PMC11218052

[27] Ares G, Antúnez L, de León C, Alcaire F, Vidal L, Natero V, et al. Even if you don’t pay attention to it, you know it’s there’: A qualitative exploration of adolescents’ experiences with digital food marketing. Appetite. 2022;176:106128. 35718311 10.1016/j.appet.2022.106128

[28] Demers-Potvin É, White M, Potvin Kent M, Nieto C, White CM, Zheng X, et al. Adolescents’ media usage and self-reported exposure to advertising across six countries: implications for less healthy food and beverage marketing. BMJ Open. 2022;12:e058913. 35589343 10.1136/bmjopen-2021-058913 PMC9121490

[29] Qutteina Y, De Backer C, Smits T. Media food marketing and eating outcomes among pre-adolescents and adolescents: A systematic review and meta‐analysis. Obes Rev. 2019;20:1708–19. 31468652 10.1111/obr.12929

[30] Boyland E, McGale L, Maden M, Hounsome J, Boland A, Angus K, et al. Association of food and nonalcoholic beverage marketing with children and adolescents’ eating behaviors and health: a systematic review and meta-analysis. JAMA Pediatr. 2022;176:e221037. 10.1001/jamapediatrics.2022.1037 PMC9062773 35499839

[31] Scully M, Wakefield M, Niven P, Chapman K, Crawford D, Pratt IS, et al. Association between food marketing exposure and adolescents’ food choices and eating behaviors. Appetite. 2012;58:1–5. 22001023 10.1016/j.appet.2011.09.020

[32] Baldwin HJ, Freeman B, Kelly B. Like and share: associations between social media engagement and dietary choices in children. Public Health Nutr. 2018;21:3210–5. 30086811 10.1017/S1368980018001866 PMC10260990

[33] Ares G, Alcaire F, Antúnez L, Natero V, de León C, Gugliucci V, et al. Exposure effects to unfamiliar food advertisements on youtube: A randomized controlled trial among adolescents. Food Qual Prefer. 2023;111:104983.

[34] Khan T, Abimbola S, Kyobutungi C, Pai M. How we classify countries and people—and why it matters. BMJ Glob Health. 2022;7:e009704. 35672117 10.1136/bmjgh-2022-009704 PMC9185389

[35] WHO. Global school-based student health survey. Uruguay 2019 fact sheet. Geneva; 2019.

[36] OPS. Alimentos y bebidas ultraprocesados en América Latina: tendencias, efecto sobre la obesidad e implicaciones para las políticas públicas. Washington; 2015.

[37] Köncke F, Berón C, Toledo C, Ceriani F, Iervolino A, Klaczko I, et al. Consumo Aparente de alimentos y Bebidas En Los Hogares uruguayos: Una Mirada a La Realidad Nacional y En Hogares Donde viven Niños Menores de 5 Años. Montevideo: Ministerio de Salud Pública; 2023.

[38] Ares G, Antúnez L, Alcaire F, Vidal L, Bove I. Listening to the voices of adolescents for the design of strategies to promote healthy eating: an exploratory study in a Latin American country. Public Health Nutr. 2021;24:5953–62. 34105451 10.1017/S1368980021002548 PMC10195589

[39] INE. Encuesta de Usos de tecnologías de La Información. Montevideo: INE; 2019.

[40] Centro de Investigaciones Económicas. Índice de Nivel socioeconómico. Montevideo: Centro de Investigaciones Económicas; 2023.

[41] R Core Team. R: A language and environment for statistical computing. 2024.

[42] Fagerland MW, Hosmer DW. How to test for goodness of fit in ordinal logistic regression models. Stata J. 2017;17:668–86.

[43] Jay M. Package ‘generalhoslem’. 2024. https://cran.r-project.org/web/packages/generalhoslem/generalhoslem.pdf.

[44] TikTok. Guardian’s guide. 2024. https://www.tiktok.com/safety/en/guardians-guide#.

[45] Instagram. About Instagram teen privacy and safety settings. 2024. https://help.instagram.com/3237561506542117#.

[46] van der Bend DLM, Jakstas T, van Kleef E, Shrewsbury VA, Bucher T. Making sense of adolescent-targeted social media food marketing: A qualitative study of expert views on key definitions, priorities and challenges. Appetite. 2022;168:105691. January 2021. 34509544 10.1016/j.appet.2021.105691

[47] Gugliucci V, Machín L, Alcaire F, Otterbring T, de León C, Natero V, et al. The content of Instagram posts featuring ultra-processed products through the lens of the heuristic-systematic model. Appetite. 2023;181:106393. 36427563 10.1016/j.appet.2022.106393

[48] Vassallo AJ, Kelly B, Zhang L, Wang Z, Young S, Freeman B. Junk food marketing on instagram: content analysis. J Med Internet Res. 2018;20:1–11. 10.2196/publichealth.9594 PMC6008515 29871854

[49] Bleakley A, Ellithorpe ME, Jordan AB, Hennessy M, Stevens R. A content analysis of sports and energy drink advertising. Appetite. 2022;174:106010. 35346764 10.1016/j.appet.2022.106010 PMC9058213

[50] Buchanan L, Yeatman H, Kelly B, Kariippanon K. A thematic content analysis of how marketers promote energy drinks on digital platforms to young Australians. Aust N Z J Public Health. 2018;42:530–1. 30370962 10.1111/1753-6405.12840

[51] Horta PM, Rodrigues FT, Dos Santos LC. Ultra-processed food product brands on Facebook pages: highly accessed by Brazilians through their marketing techniques. Public Health Nutr. 2018;21:1515–9. 29444739 10.1017/S1368980018000083 PMC10261659

[52] Freeman B, Kelly B, Baur L, Chapman K, Chapman S, Gill T, et al. Digital junk: food and beverage marketing on Facebook. Am J Public Health. 2014;104:e56–64. 25322294 10.2105/AJPH.2014.302167 PMC4232106

[53] Potvin Kent M, Bagnato M, Amson A, Remedios L, Pritchard M, Sabir S, et al. #junkfluenced: the marketing of unhealthy food and beverages by social media influencers popular with Canadian children on youtube, Instagram and TikTok. Int J Behav Nutr Phys Activity. 2024;21:37. 10.1186/s12966-024-01589-4 PMC11010392 38605322

[54] Winzer E, Naderer B, Klein S, Lercher L, Wakolbinger M. Promotion of food and beverages by German-Speaking influencers popular with adolescents on tiktok, YouTube and Instagram. Int J Environ Res Public Health. 2022;19:10911. 36078625 10.3390/ijerph191710911 PMC9518047

[55] Elliott C, Truman E, Aponte-Hao S. Food marketing to teenagers: examining the power and platforms of food and beverage marketing in Canada. Appetite. 2022;173:105999. 35292304 10.1016/j.appet.2022.105999

[56] Murphy G, Corcoran C, Tatlow-Golden M, Boyland E, Rooney B, See. Like, share, remember: adolescents’ responses to Unhealthy-, Healthy- and Non-Food advertising in social media. Int J Environ Res Public Health. 2020;17:2181. 32218252 10.3390/ijerph17072181 PMC7177346

[57] Kelly B, Bosward R, Freeman B. Australian children’s exposure to, and engagement with, Web-Based marketing of food and drink brands: Cross-sectional observational study. J Med Internet Res. 2021;23:e28144. 34255675 10.2196/28144 PMC8314155

[58] Kelly B, Vandevijvere S, Ng S, Adams J, Allemandi L, Bahena-Espina L, et al. Global benchmarking of children’s exposure to television advertising of unhealthy foods and beverages across 22 countries. Obes Rev. 2019;20:116–28. 30977265 10.1111/obr.12840 PMC6988129

[59] Ares G, Alcaire F, Gugliucci V, Machín L, de León C, Natero V, et al. Colorful candy, teen vibes and cool memes: prevalence and content of Instagram posts featuring ultra-processed products targeted at adolescents. Eur J Mark. 2023. 10.1108/EJM-12-2022-0899.

[60] Federal Trade Commission. A review of food marketing to children and adolescentes. Follow-up report. 2012.

[61] Schneble CO, Favaretto M, Elger BS, Shaw DM. Social media terms and conditions and informed consent from children: ethical analysis. JMIR Pediatr Parent. 2021;4:e22281. 33885366 10.2196/22281 PMC8103294

[62] Castronuovo L, Guarnieri L, Tiscornia MV, Allemandi L. Food marketing and gender among children and adolescents: a scoping review. Nutr J. 2021;20:52. 34099000 10.1186/s12937-021-00706-4 PMC8186097

[63] Kelly B, Boyland E, Tatlow-Golden M, Christiansen P. Testing a conceptual hierarchy of effects model of food marketing exposure and associations with children and adolescents’ diet-related outcomes. Public Health Nutr. 2024;27:e10. 10.1017/S1368980023002616 PMC10830363 38058182

[64] Hendrikse JJ, Cachia RL, Kothe EJ, McPhie S, Skouteris H, Hayden MJ. Attentional biases for food cues in overweight and individuals with obesity: a systematic review of the literature. Obes Rev. 2015;16:424–32. 25752592 10.1111/obr.12265

[65] Folkvord F, Veling H, Hoeken H. Targeting implicit approach reactions to snack food in children: effects on intake. Health Psychol. 2016;35:919–22. 27505216 10.1037/hea0000365

[66] Sacks G, Looi ESY. The advertising policies of major social media platforms overlook the imperative to restrict the exposure of children and adolescents to the promotion of unhealthy foods and beverages. Int J Environ Res Public Health. 2020;17:1–11. 10.3390/ijerph17114172 PMC7312784 32545343

[67] WHO. Policies to protect children from the harmful impact of food marketing: WHO guideline. Geneva: WHO; 2023. 37440677

[68] Boyland E, McGale L, Maden M, Hounsome J, Boland A, Jones A. Systematic review of the effect of policies to restrict the marketing of foods and non-alcoholic beverages to which children are exposed. Obes Rev. 2022;23:e13447. 35384238 10.1111/obr.13447 PMC9541016

[69] Ares G, Antúnez L, Alcaire F, Natero V, Otterbring T. Is this advertisement designed to appeal to you? Adolescents’ views about Instagram advertisements promoting ultra-processed products. Public Health Nutr. 2024;27:e96. 38449441 10.1017/S1368980024000533 PMC10993065

[70] Powell T, Gheera M, Foster D, Balogun B, Conway L. The health and care bill [Bill 140 of 2021-22]. London: Department of Health & Social Care; 2021. https://researchbriefings.files.parliament.uk/documents/CBP-9232/CBP-9232.pdf.

[71] Bulger M, Davison P. The promises, challenges, and futures of media literacy. J Media Lit Educ. 2018;10:1–21.37077620

[72] Cullerton K, Donnet T, Lee A, Gallegos D. Effective advocacy strategies for influencing government nutrition policy: a conceptual model. Int J Behav Nutr Phys Activity. 2018;15:83. 10.1186/s12966-018-0716-y PMC6119246 30170610

[73] Russell C, Lawrence M, Cullerton K, Baker P. The political construction of public health nutrition problems: a framing analysis of parliamentary debates on junk-food marketing to children in Australia. Public Health Nutr. 2020;23:2041–52. 31948503 10.1017/S1368980019003628 PMC10200508

[74] Voorberg WH, Bekkers VJJM, Tummers LG. A systematic review of Co-Creation and Co-Production: embarking on the social innovation journey. Public Manage Rev. 2015;17:1333–57.

[75] Packer J, Croker H, Goddings A-L, Boyland EJ, Stansfield C, Russell SJ, et al. Advertising and young people’s critical reasoning abilities: systematic review and meta-analysis. Pediatrics. 2022;150:e2022057780. 10.1542/peds.2022-057780 PMC9724173 36377381

[76] Savage JS, Mitchell DC, Smiciklas-Wright H, Symons Downs D, Birch LL. Plausible reports of energy intake May predict body mass index in Pre-Adolescent girls. J Am Diet Assoc. 2008;108:131–5. 18155999 10.1016/j.jada.2007.10.006 PMC2531147

[77] Jones L, Ness A, Emmett P. Misreporting of energy intake from food records completed by adolescents: associations with sex, body image, nutrient, and food group intake. Front Nutr. 2021;8:749007. 10.3389/fnut.2021.749007 PMC8710752 34966768

