# Supplementary material for: Associations between exposure to digital food marketing and food consumption in adolescence: a cross-sectional study in an emerging country
Source: BMC Public Health. 2025 Sep 30;25:3158. doi: 10.1186/s12889-025-24443-z (PMC12486551; doi:10.1186/s12889-025-24443-z)
Supplement: Supplementary file 1 — Supplementary Material 1 [file 12889_2025_24443_MOESM1_ESM.pdf]

Supplementary material

Figure 1. English translation of the questionnaire.

Espacio Interdisciplinario
Universidad de la República
Uruguay

SURVEY ON FOOD ADVERTISING IN SOCIAL MEDIA 2024

**Participant number:**
**Institution number:**

In the following section, we are going to ask you a few questions about advertisements of foods and beverages

1. Have you seen advertisements of foods and beverages on social media or websites in the last week?

- Yes, more than once a day.................... ☐
- Yes, once a day....................................... ☐
- Yes, several times a week........................ ☐
- Yes, once in the week............................... ☐
- No, I haven't seen any advertisement.... ☐

2. What advertisements of food and beverages do you recall seeing on social media?

☐

3. In which of the following media do you remember seeing any food or beverage advertisements in the last week?

Instagram	Yes ☐	No ☐
TikTok	Yes ☐	No ☐
Facebook	Yes ☐	No ☐
YouTube	Yes ☐	No ☐
Snapchat	Yes ☐	No ☐
Twitter	Yes ☐	No ☐
Web browsing	Yes ☐	No ☐
Twitch	Yes ☐	No ☐

4. Do you remember seeing any advertisement of the following food or beverages on social media or websites in the last week?

Soft drinks	Yes ☐	No ☐
Energy drinks	Yes ☐	No ☐
Flavoured water	Yes ☐	No ☐
Bottled juices or powdered drinks	Yes ☐	No ☐
Cookies or crackers	Yes ☐	No ☐
Alfajores	Yes ☐	No ☐
Flavoured milk, yogurt, or milk desserts	Yes ☐	No ☐
Chocolates or confectionary	Yes ☐	No ☐
Ice-cream	Yes ☐	No ☐
Cold-cuts or sausages (e.g., ham, salami, sausages, hot dogs)	Yes ☐	No ☐
Breakfast cereals or cereal bars	Yes ☐	No ☐
Bakery products (e.g., croissants, donuts, pastries)	Yes ☐	No ☐
Savoury snacks (e.g., potato chips, Doritos, Cheetos)	Yes ☐	No ☐
Condiments (e.g., ketchup, mustard, mayonnaise)	Yes ☐	No ☐
Soups or broths	Yes ☐	No ☐
Marmalades or dulce de leche	Yes ☐	No ☐
Food eaten in fast food restaurants (e.g., burgers, pizza, French fries)	Yes ☐	No ☐

In the following section you will see a list of foods and beverages. We will ask you to indicate how often you have eaten or drunk them last week.

5. How many days last week did you eat **fruits**?

0 days	1 day	2 to 3 days	4 to 6 days	7 days
☐	☐	☐	☐	☐

6. How many days last week did you eat **vegetables**?

0 days	1 day	2 to 3 days	4 to 6 days	7 days
☐	☐	☐	☐	☐

7. How many days last week did you eat **beef, chicken or pork**?

0 days	1 day	2 to 3 days	4 to 6 days	7 days
☐	☐	☐	☐	☐

8. How many days last week did you eat **fish**?

0 days	1 day	2 to 3 days	4 to 6 days	7 days
☐	☐	☐	☐	☐

9. How many days last week did you drink **milk or eat cheese**?

0 days	1 day	2 to 3 days	4 to 6 days	7 days
☐	☐	☐	☐	☐

10. How many days last week did you drink **soft drinks**?

0 days	1 day	2 to 3 days	4 to 6 days	7 days
☐	☐	☐	☐	☐

11. How many days last week did you drink **energy drinks**?

0 days	1 day	2 to 3 days	4 to 6 days	7 days

12. How many days last week did you drink **flavoured water**?

0 days	1 day	2 to 3 days	4 to 6 days	7 days

13. How many days last week did you drink **bottled juices or powdered drinks**?

0 days	1 day	2 to 3 days	4 to 6 days	7 days
☐	☐	☐	☐	☐

14. How many days last week did you eat **cookies or crackers**?

0 days	1 day	2 to 3 days	4 to 6 days	7 days
☐	☐	☐	☐	☐

15. How many days last week did you eat **alfajores**?

0 days	1 day	2 to 3 days	4 to 6 days	7 days
☐	☐	☐	☐	☐

16. How many days last week did you drink **flavoured milk or yogurt** or eat **dairy desserts**?

0 days	1 day	2 to 3 days	4 to 6 days	7 days
☐	☐	☐	☐	☐

17. How many days last week did you eat **chocolates or candies**?

0 days	1 day	2 to 3 days	4 to 6 days	7 days
☐	☐	☐	☐	☐

18. How many days last week did you eat **ice-cream**?

0 days	1 day	2 to 3 days	4 to 6 days	7 days
☐	☐	☐	☐	☐

19. How many days last week did you eat **cold cuts or sausages** (e.g., ham, salami, sausages, hot dogs)?

0 days	1 day	2 to 3 days	4 to 6 days	7 days
☐	☐	☐	☐	☐

20. How many days last week did you eat **breakfast cereals or cereal bars**?

0 days	1 day	2 to 3 days	4 to 6 days	7 days
☐	☐	☐	☐	☐

21. How many days last week did you eat **bakery products** (e.g., croissants, donuts, pastries)?

0 days	1 day	2 to 3 days	4 to 6 days	7 days
☐	☐	☐	☐	☐

23. How many days last week did you eat **savoury snacks** (e.g., potato chips, Doritos, Cheetos)?

0 days	1 day	2 to 3 days	4 to 6 days	7 days
☐	☐	☐	☐	☐

24. How many days last week did you eat **ketchup, mustard or mayonnaise**?

0 days	1 day	2 to 3 days	4 to 6 days	7 days
☐	☐	☐	☐	☐

25. How many days last week did you eat or ordered **at a fast-food restaurant** (e.g., burgers, pizza, French fries)?

0 days	1 day	2 to 3 days	4 to 6 days	7 days
☐	☐	☐	☐	☐

The following questions are related to social media and TV

26. On a normal weekday, how much time do you use **Instagram**?

I don't use	Less than 15 minutes	15 to 30 minutes	30 minutes to 1 hour	1 to 2 hours	2 to 3 hours	3 to 4 hours	More than 4 hours
☐	☐	☐	☐	☐	☐	☐	☐

27. On a normal weekday, how much time do you use **TikTok**?

I don't use	Less than 15 minutes	15 to 30 minutes	30 minutes to 1 hour	1 to 2 hours	2 to 3 hours	3 to 4 hours	More than 4 hours
☐	☐	☐	☐	☐	☐	☐	☐

28. On a normal weekday, how much time do you use **Facebook**?

I don't use	Less than 15 minutes	15 to 30 minutes	30 minutes to 1 hour	1 to 2 hours	2 to 3 hours	3 to 4 hours	More than 4 hours
☐	☐	☐	☐	☐	☐	☐	☐

29. On a normal weekday, how much time do you use **Twitter**?

I don't use	Less than 15 minutes	15 to 30 minutes	30 minutes to 1 hour	1 to 2 hours	2 to 3 hours	3 to 4 hours	More than 4 hours
☐	☐	☐	☐	☐	☐	☐	☐

30. On a normal weekday, how much time do you use **YouTube**?

I don't use	Less than 15 minutes	15 to 30 minutes	30 minutes to 1 hour	1 to 2 hours	2 to 3 hours	3 to 4 hours	More than 4 hours
☐	☐	☐	☐	☐	☐	☐	☐

31. On a normal weekday, how much time do you use **website browsing**?

I don't use	Less than 15 minutes	15 to 30 minutes	30 minutes to 1 hour	1 to 2 hours	2 to 3 hours	3 to 4 hours	More than 4 hours
☐	☐	☐	☐	☐	☐	☐	☐

32. On a normal weekday, how much time do you use **Snapchat**?

I don't use	Less than 15 minutes	15 to 30 minutes	30 minutes to 1 hour	1 to 2 hours	2 to 3 hours	3 to 4 hours	More than 4 hours
☐	☐	☐	☐	☐	☐	☐	☐

33. On a normal weekday, how much time do you use **Twitch**?

I don't use	Less than 15 minutes	15 to 30 minutes	30 minutes to 1 hour	1 to 2 hours	2 to 3 hours	3 to 4 hours	More than 4 hours
☐	☐	☐	☐	☐	☐	☐	☐

34. On a normal weekday, how much time do you **watch TV** (including cable TV, Netflix, Disney+ or other platforms)?

I don't use	Less than 15 minutes	15 to 30 minutes	30 minutes to 1 hour	1 to 2 hours	2 to 3 hours	3 to 4 hours	More than 4 hours
☐	☐	☐	☐	☐	☐	☐	☐

The last questions are about yourself

37. What is your gender?

Feminine.............................. ☐
Masculine.............................. ☐
Other.................................... ☐

36. How old are you?

Age ___________________________

37. In which neighbourhood do you live?

Neighbourhood ______________________

You have reached the end of the questionnaire.

Thank you very much!

Table 1. Results of the ordinal logistic regressions analysing the association between consumption frequency of specific ultra-processed product categories and self-reported exposure to an advertisement of the food category on digital media in the previous week and total social media use (hours), while controlling for gender, age range, socio-economic status and TV watching time. Results of the Lipsitz goodness of fit and Pulkstenis-Robinson chi-squared tests are also shown.

**Food group**	**Exposure to a food advertisement** (Yes vs. No)	**Total social media use** (hours)	**Socio-economic status** (Low vs. High)	**Socio-economic status** (Medium vs. High)	**Age range** (15.19 vs. 11-14)	**Gender** (Male vs. Female)	**TV use** (hours)	**Lipsitz test**	**Pulkstenis-Robinson test**
Soft drinks	**<0.001**	**<0.001**	0.17	0.69	0.169	0.255	**<0.001**	0.886	0.499
Energy drinks	**<0.001**	**<0.001**	0.35	0.73	**0.029**	**0.022**	0.904	0.827	0.148
Flavoured water	**<0.001**	0.241	0.494	0.489	0.301	0.718	0.071	0.916	0.094
Bottled juices or powdered drinks	**0.016**	**0.003**	0.106	0.072	**<0.001**	0.292	0.057	0.52	0.719
Savory snacks	**<0.001**	**<0.001**	0.565	0.648	**0.028**	0.705	0.077	0.304	0.669
Bakery products	**<0.001**	**0.001**	0.068	**0.047**	**0.549**	**0.443**	**<0.001**	0.256	0.407
Cookies or crackers	0.988	0.150	0.988	0.617	0.129	0.637	**0.028**	0.619	0.576
Alfajores	**<0.001**	**<0.001**	0.413	0.704	0.093	0.093	0.08	0.275	0.762
Breakfast cereals or cereal bars	**<0.001**	0.487	**0.019**	**0.003**	0.63	0.936	0.234	0.478	0.516
Flavoured milk, yogurt or dairy deserts	**<0.001**	**<0.001**	0.629	0.114	**<0.001**	0.586	0.767	0.982	0.186
Ice-cream	**0.006**	0.098	**0.008**	**0.005**	0.215	0.883	0.956	0.63	0.623
Chocolates or confectionary	**<0.001**	**0.004**	0.197	**0.032**	0.098	**<0.001**	0.052	0.645	0.567
Cold cuts and sausages	**0.002**	**0.047**	**0.002**	0.328	**0.752**	**<0.001**	**0.009**	0.241	0.846
Ketchup or mayonnaise	**<0.001**	**<0.001**	0.702	0.746	0.472	**0.011**	**0.015**	0.99	0.513
Fast food	**0.050**	**<0.001**	0.414	**<0.001**	0.345	**0.011**	0.489	0.757	**0.002**

Notes: Participants with missing data (n=24) or whose self-reported gender identities (n=9) different from males and females were not considered in the model. The reference in the model for consumption frequency was '0 days' compared to '1 day', and '2-3 days', '4-6 days' and '7 days'. p-values highlighted in bold are significant at 0.05.

Table 2. Results of the ordinal logistic regressions analysing the association between consumption frequency of specific food groups and self-reported exposure to digital food marketing in the week prior to the survey and total social media use (hours), while controlling for gender, age range, socio-economic status and TV watching time. Results of the Lipsitz goodness of fit and Pulkstenis-Robinson chi-squared tests are also shown.

**Food group**	**Exposure to a food advertisement** (Yes vs. No)	**Total social media use** (hours)	**Socio-economic status** (Low vs. High)	**Socio-economic status** (Medium vs. High)	**Age range** (15.19 vs. 11-14)	**Gender** (Male vs. Female)	**TV use** (hours)	**Lipsitz test**	**Pulkstenis-Robinson test**
Fruits	0.311	**<0.001**	**<0.001**	**<0.001**	0.613	**<0.001**	0.636	0.171	0.16
Vegetables	0.298	**0.009**	0.088	**0.02**	**0.005**	**<0.001**	0.466	0.052	0.101
Meats	0.646	0.169	0.102	0.795	**0.039**	**<0.001**	0.466	0.416	0.274
Fish	0.928	0.206	0.074	0.053	0.73	**<0.001**	0.654	0.910	**<0.001**

Notes: Participants with missing data (n=24) or whose self-reported gender identities (n=9) different from males and females were not considered in the model. The reference in the model for consumption frequency was '0 days' compared to '1 day', and '2-3 days', '4-6 days' and '7 days'. p-values highlighted in bold are significant at 0.05.
